# SiRNA targeting *EGFR* effectively prevents posterior capsular opacification after cataract surgery

**Published:** 2011-08-31

**Authors:** Wan-Rong Huang, Xing-Xing Fan, Xin Tang

**Affiliations:** Tianjin Eye Hospital, Tianjin Medical University, Tianjin, China

## Abstract

**Purpose:**

We investigated the effect of epidermal growth factor receptor (*EGFR*) siRNA on human lens epithelium (HLE) cells and the development of posterior capsular opacity (PCO).

**Methods:**

We designed *EGFR* siRNA and used it to knockdown the expression of *EGFR* in HLE cells. Cell proliferation was examined by 3-(4,5-Dimethylthiazol-2-yl)-2,5-diphenyltetrazolium bromide (MTT), cell growth curve assay and cell cycle analysis. Next, we selected an adaptable concentration of recombinant epidermal growth factor (EGF) for stimulating the growth of HLE cells to further test the suppressive effect of siRNA. At last, we established the model of PCO in rats to further investigate whether knocking down *EGFR* would prevent the progression of PCO in vivo.

**Results:**

The cell proliferation of *EGFR* siRNA group was apparently inhibited no matter in short or long term and cell cycle was arrested in G_1_ phase. Over expression EGF cannot rescue the inhibition of *EGFR* siRNA on HLE cells and the proliferation activity in HLE cells greatly decreased when EGF-EGFR signal pathway blockaded. In vivo experiments, the extent of PCO of *EGFR* siRNA group is much lower than the control group.

**Conclusions:**

Our results demonstrate that *EGFR* siRNA can effectively inhibit the progression of PCO. Thus, siRNA targeting *EGFR* may provide a totally new way for preventing PCO or even cataract.

## Introduction

Posterior capsule opacification (PCO) is the most frequent complication and the primary reason of visual decrease after extracapsular cataract surgery. While most patients benefit from this treatment initially, within 5 years of surgery about 20%–40% suffers a secondary loss of vision because of posterior capsule opacification (PCO), also known as after-cataract [1].

Because the residual lens epithelial cells at the equator and under the anterior lens capsule proliferate and migrate onto the posterior capsule and undergo epitheliale-mesenchymal transition (EMT). This results in the formation of fibroblasts and myofibroblasts, as well as the formation of extracellular cell matrix (ECM), and finally, the PCO [2-4].

Epidermal growth factor (EGF) is a growth factor that plays an important role in the regulation of cell growth, proliferation, and differentiation by binding to its receptor EGFR. The epidermal growth factor receptor is a member of the ErbB family of receptors, a subfamily of four closely related receptor tyrosine kinases: EGFR (ErbB-1), Her2 (human epidermal growth factor receptor 2)/c-neu (ErbB-2), Her 3 (ErbB-3) and Her 4 (ErbB-4) [5]. Mutations that lead to EGFR overexpression or over activity have been associated with several cancers [6]. Especially, mutations, amplifications or mis-regulations of EGFR or family members are implicated in about 30% of all epithelial cancers [7]. Some factors such as injury may enhance the expression of EGFR [8].

The present study was undertaken to investigate the hypothesis that EGFR exerts a critical role on PCO formation by promoting the survival of abnormal cells with PCO-like characteristics and the blockage of expression of *EGFR* in HLE cells may reduce the development of PCO.

## Methods

### Cell culture

HLE cells were purchased from the ATCC (Manassas, VA), grown in RPMI-1640 culture medium, supplemented with 20% premium FBS, 50 U/ml of penicillin, 50 μg/ml streptomycin, and 50 μg/ml gentamycin. Cells were maintained in a humidified 37 °C atmosphere of ambient air/5% CO2.

### The construction of siRNA expression system

To construct the siRNA expression vector of *EGFR* (pSilencer-EGFR), a 74 bp double strand si-*EGFR* was obtained by annealing single strand EGFR-Top line, 5′-GGA TCC CGT GGA GCG AAT TCC TTT GGA ATT CAA GAG ATT CCA AAG GAA TTC GCT CCA CTT TTT TGG AAA AGC TT-3′; and EGFR-Bottom line, 5′-AAG CTT TTC CAA AAA AGT GGA GCG AAT TCC TTT GGA ATC TCT TGA ATT CCA AAG GAA TTC GCT CCA CGG GAT CC-3′; Then the double strand was cloned into vector pSilencer 2.1-neo (Ambion, Austin, TX). Annealing was performed as: 95 °C for 5 min and room temperature 2 h.

### SiRNA transfection

Transient transfection of siRNA expression vectors was performed using Lipofectamine transfection reagent 2000 (Invitrogen, Carlsbad, CA), according to the manufacturer’s protocol. HLE cells (5×10^5^ cells per well) were seeded in a 24-well plate or 1.5×10^6^ cells were seeded in 25 ml culture flask. After 16 h at about 60% confluence, the cells were transfected with *EGFR* siRNA (1 μg/well, 4 ×g/flask). Four h after transfection, full culture medium, without antibiotics, was added.

### MTT assay

HLE cells (8,000 cell/well) in logarithmic growth phase were cultured in 96-well flat-bottomed plates in a triplicate pattern. Forty-eight h after transfection, 20 μl 3-(4,5-Dimethylthiazol-2-yl)-2,5-diphenyltetrazolium bromide (MTT) solution (5 mg/ml) was added to each well which have already contained 100 μl culture media and incubated for 4 h at 37 °C. Then 200 μl of DMSO was added to each well and the plate was vortexed for 10 min at 37 °C. Finally, the optical density value of each well was measured at 570 nm.

### Cell growth curve assay

To determine the proliferative ability of cells, 3×10^4^ cells were plated in 24-well plates (three replicates for each time point), and the number of cells was counted everyday after plating. The growth assay was performed over a period of 8 days, at which time the cells were confluent.

### Cell cycle assay

Cells (1×10^6^) were plated in 60 mm culture dishes. After an overnight incubation for the cells to adhere, cells were treated with *EGFR* siRNA and non-silencing siRNA. After incubation for 48 h, the cells were then washed twice with cold phosphate buffered saline (PBS), detached with 0.25% trypsin-EDTA and pelleted. The pellet was suspended in cold PBS and the cells were fixed in a final concentration of 70% ethanol for 1 h at 4 °C. The cells were washed with cold PBS and incubated with 100 μg/ml RNase A for 15 min at 37 °C. Nuclei were stained with 50 mg/ml propidium iodide (PI; Sigma-Aldrich, St. Louis, MO) for 30 min at 37 °C in the dark. Samples were analyzed by flow cytometry. For flow cytometric evaluation of cell cycle, 10,000 events corrected for debris and aggregate were analyzed for each sample. The proliferation index (PI) was calculated as follows: PI=(S+G_2_)/G_1_.The assay was performed in three replicates.

### Real-time PCR

Forty-eight h after transfection, total RNA was extracted. Total mRNAs were reverse transcribed by oligodT primers. The housekeeping gene β-actin (*ACTB*) was considered as an endogenous control target gene and controls were treated with the same conditions and analyzed by real-time PCR. Real-time PCR was performed as described in the method of SYBR pre-Mix kits (TakaRa, Dalian, China).

### Western blot

Forty-eight h after transfection, HLE cells were treated with radio immunoprecipitation assay (RIPA) lysis buffer containing 1% protease inhibitors (Roche, Basel, Switzerland) and proteins were harvested. Equal amounts of protein were electrophoresed on a 10% SDS–PAGE gel and then electro transferred to a nitrocellulose membrane (Millipore, Bedford, MA). The membrane was blocked with 5% nonfat milk for 2 h. Then the membranes were incubated with rabbit anti-human EGFR (1:200; Santa Cruz Biotechnology, Santa Cruz, CA) or GAPDH antibody (1:1000; Santa Cruz Biotechnology) in 5% nonfat milk overnight, respectively. The membranes were incubated by a goat anti-rabbit antibody (1:1000; Santa Cruz Biotechnology) for 90 min at room temperature. Protein expression was assessed by enhanced chemiluminescence and exposure to chemiluminescent film.

### Aminal experiment, siRNA transfection in vivo

Forty-eight Sprague Dawley (SD) rats were provided by Tianjin medical university, Tianjin, China, animal center. All studies were performed in accordance with the ARVO Statement for the Use of Animals in Ophthalmic and Vision Research and all animal research protocols were approved by the Tianjin Medical University for Comparative Medicine. The right eye of each rat was operated for extracapsluar cataract extraction in all the animals and the rats were divided into 2 groups at random. Abdominal injection of Aminazine (25 mg/kg) and Ketamine (50 mg/kg) was used for general anesthesia. After routine preoperation preparation, make a incision in the clear cornea with 15° paracentesis knife. After injecting Healon, make a 3–3.5 mm anterior continue circular capsulotomy with discission needle, then enlarge the corneal incision to 120° parallel corneal limbus, revolve lens nucleus into anterior chamber after water separation with BSS (balanced salt solution) and extract it, then aspirate the residual cortex and irrigate anterior chamber with BSS. Close the incision with 10–0 nylon. Then injected 100 μl transfection mixture into each capsule bag. The control group was given plasmid pSilencer 2.1 (5 μg), and the siRNA group was treated with pSilencer-EGFR (5 μg). Eight rats from each group were separately sacrificed at 1, 7, and 14 days after surgery and 4 eyes were enucleated and processed for light microscopy and histology analysis, and the other 4 lens capsules were prepared for western-blot.

### Histology

For paraffin embedded samples, each tissue was sectioned (6 μm) serially perpendicular to the lens capsule and slides was stained with hematoxylin and eosin. The slides were observed under microscope and photographed. Then two slides of each group at each time point were selected to count the number of cells. Cells in 9 random fields of view at 200× magnification were counted and expressed as the average number of cells/field of view. All assays were performed in triplicate.

### Statistical analysis

Data are expressed as the mean±standard deviation (SD), and a p<0.05 was considered statistically significant by the Students-Newman-Keuls test.

## Results

### SiRNA significantly knock downed the expression of *EGFR*

**Figure 1:**
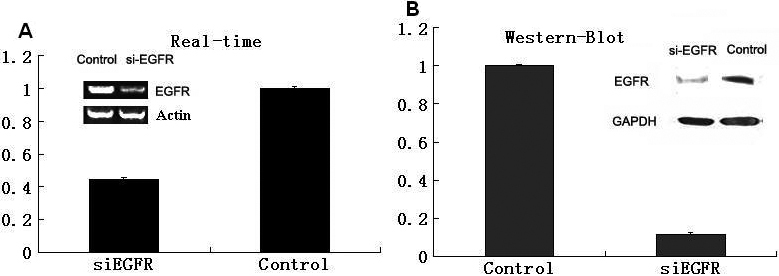
SiRNA significantly knock downed the expression of *EGFR* in HLE cells. **A**: Real-time PCR was performed to detect the EGFR level in HLE cells treated with *EGFR* siRNA or non-silencing control siRNA. **B**: Western-Blot was also performed to detect the EGFR level in these two groups. p<0.05.

To test the efficiency of siRNA, the constructed siRNA expression vector was transfected into HLE cells. After 48 h, total RNA and protein were extracted from the cells, and then Real-time and western-blot were applied to test the changes of *EGFR* expression. From Figure 1, the results suggested that the expression of *EGFR* on both the RNA and protein level were effectively suppressed by siRNA. *EGFR* siRNA treated group was only 45 percent on RNA level and 10 percent on protein level of the control group which is treated with non-silencing siRNA.

### The effect of siRNA on HLE cell growth

**Figure 2:**
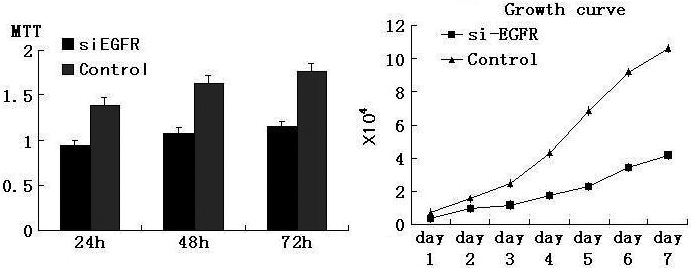
MTT and growth curve array was used to determine the effect of *EGFR* siRNA on HLE cell proliferation activity. p<0.05.

Since the siRNA can obviously knockdown the expression of *EGFR*, we intended to determined whether it can affect the growth ability of HLE cells. MTT and cell growth curve assay were used to test the changes of cell growth ability in short-term and long-term culture, respectively (Figure 2). From the figure, we can determine that no matter whether short-term or long-term culture was used, the proliferation of cells in the siRNA group was largely suppressed when compared to the control group. This tendency became more apparent as time increased.

### SiRNA of *EGFR* arrested the G_1_ phase transition

**Figure 3:**
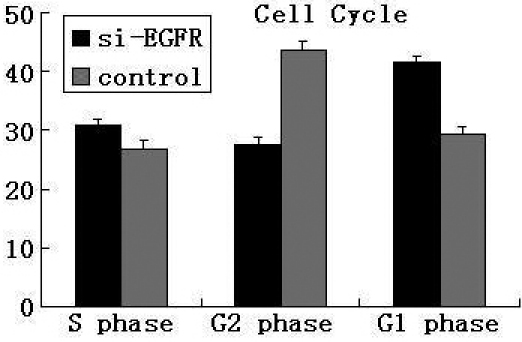
Cell cycle test was applied to check whether *EGFR* siRNA affect the proliferation of HLE cells by arresting the cell cycle. Cells treated with siRNA of *EGFR* were G_1_ phase arrested. p<0.05.

To explore whether the inhibition of HLE cells growth was due to the alteration in cell cycle progression, we performed fluorescence-activated cell sorting (FACS) analysis. Interestingly, in the *EGFR* siRNA treated group, the percentage of cells in G_1_ phase increased to 41.6%, as compared to the control, which had only 29.5% of cells in G_1_ phase. The percentage of *EGFR* siRNA cells in G_2_ phase decreased to 27.5%, as compared to 43.7% in the control group (Figure 3). The proliferation index of *EGFR* siRNA treated cells was 140.4% as compared to 239.0% in controls. These results indicate that *EGFR* plays an important role in the transition of the cell cycle and affecting the growth of HLE cells.

### The role of EGF-EGFR signaling pathway in HLE cell proliferation

**Figure 4:**
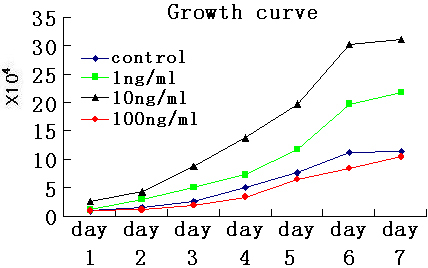
Selecting a most suitable concentration of EGF for HLE cells growth. p<0.05.

Since *EGFR* plays a critical role in the proliferation of epidermal cells, we tested the extent of the affect on growth characteristics of HLE cells. We used three different EGF concentrations to treat cells and observed their effects on cells growth. From Figure 4, we can make a conclusion that in a certain range, the proliferation ability of cells is rising following the increase of EGF concentration, but if the range was exceeded, it may exert an opposing effect. We choose the most adaptable concentration of recombinant EGF (10 ng/ml) to simulate the stimulus by overexpressing EGF to further examine the effect of siRNA.

**Figure 5:**
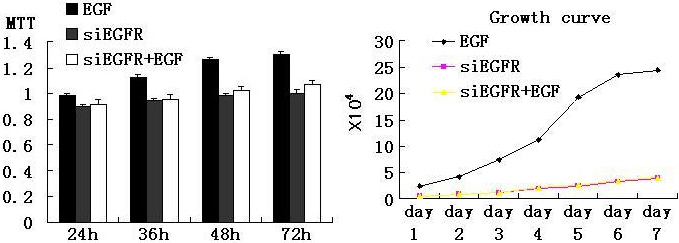
Using the selected concentration of EGF to further test the inhibitory effect of *EGFR* siRNA on cells. MTT and growth curve array were applied. Compared to the control group, it can be seen that the *EGFR* siRNA group was almost unchanged when co-supplied with EGF. p<0.05.

Compared to the control group, it can be said that the *EGFR* siRNA group was almost unchanged when co-supplied with EGF (Figure 5). Therefore, in this step, we confirmed that even spurred by EGF, siRNA of *EGFR* can stably inhibit the growth of HLE cells in vitro.

### In vivo siRNA efficiently inhibits the progression of PCO

**Figure 6:**
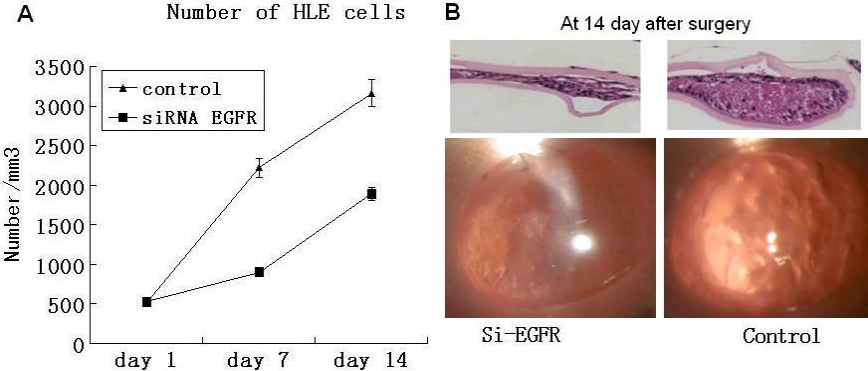
In vivo experiments demonstrated that siRNA of *EGFR* can efficiently inhibit the progression of PCO. **A**: The cell number of HLE. Cells in 9 random fields of view at 200×magnification were counted and expressed as the average number of cells/field of view, p<0.05. **B**: Slides were stained with hematoxylin and eosin.

Finally, we investigated the inhibition of *EGFR* siRNA on PCO progression in vivo. From Figure 6A,B, although we can see that the number of HLE cells increased in both groups, the increase of the control group is more than twice as that of *EGFR* siRNA treated group. And the gap between two groups became more and more obvious as time increased.

**Figure 7:**
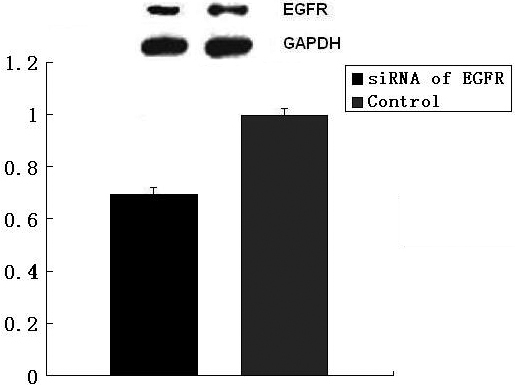
Western-blot tests the inhibitory effect of *EGFR* siRNA in vivo on HLE cells. p<0.05.

As for the increase of cells in the siRNA group, probably poor transfection efficiency in vivo is responsible. In Figure 7, the result of western blot shows that the knockdown efficiency of EGFR is about 40% when compared to the control group, which is lower than in vitro. So we believe that if we can find a new way to enhance the efficiency of EGFR, better results will be available.

## Discussion

PCO puts a great threat on maintaining normal visual function after cataract surgery [9,10]. The incidence of PCO is 30%–50% in adult, and 100% in children [11]. Though the reason of PCO has been known for some time, the mechanism of it is yet not clearly understood. A large number of studies have been taken to explore an efficient way for getting rid of this complication.

The EGFR signaling pathway has an important role in cell proliferation, especially epithelial cells. Many diseases have been reported to be closely related with the deregulation of EGFR signaling [12-14].

After activated by binding of its specific ligands, including epidermal growth factor and transforming growth factor α (TGF-α), EGFR can stimulate the intrinsic protein-tyrosine kinase activity of the receptor. The tyrosine kinase activity, in turn, initiates a signal transduction cascade that results in a variety of biochemical changes within the cell – a rise in intracellular calcium levels, increased glycolysis and protein synthesis, and increases in the expression of certain genes including *EGFR* – that ultimately lead to DNA synthesis and cell proliferation [15].

In our study, we hypothesized that *EGFR* has a vital role in the process of PCO. The morphological and immune properties of eyes make it suitable for RNAi treatment [16]. So we used RNAi and designed a series of experiments in vitro or in vivo to test our assumption.

The results of MTT and cells growth curve assay is surprisingly similar in that *EGFR* siRNA can stably suppress the proliferation of HLE cells, with or without the stimulus of EGF. And in vivo experiments, we directly gave a transfection mixture to the capsular bag of rats immediately after surgery. Maybe transfection efficiency was much lower than in vitro, but even so, the proliferation of HLE cells in the bag was significantly inhibited by siRNA. Based on these results, we probably can make such a conclusion that *EGFR* is an effective target of PCO, and siRNA of *EGFR* can be a potential drug for preventing it.

However, this study may not completely remove the problem of PCO. First of all, HLE cells also express other various kinds of growth factors and receptors in the cell membrane, and these growth factor and its receptors composing signaling pathway can enhance the proliferation, differentiation, and collagen fiber excretion [17]. For example, TGF-β was reported in many articles to be closely related with HLE cells [18-23].

Second, as we can see from the data of our studies, the efficiency of siRNA in vivo is not as high as in vitro experiments. So, if we can enhance the transfection efficiency of siRNA in vivo, we believe that a much satisfying result could be obtained. Perhaps the Lentiviral expression system could be applied to infect HLE cells in capsular bag to get desirable results.

In summary, siRNA of *EGFR* seems to be a potential therapy of PCO which might be developed into a drug in the future. And due to the instability of transient transfection, a virus expression system may provide an effective solving solution.
